# Novel Template Plasmids pCyaA’-Kan and pCyaA’-Cam for Generation of Unmarked Chromosomal *cyaA*’ Translational Fusion to T3SS Effectors in *Salmonella*

**DOI:** 10.3390/microorganisms9030475

**Published:** 2021-02-25

**Authors:** Paulina A. Fernández, Marcela Zabner, Jaime Ortega, Constanza Morgado, Fernando Amaya, Gabriel Vera, Carolina Rubilar, Beatriz Salas, Víctor Cuevas, Camila Valenzuela, Fernando Baisón-Olmo, Sergio A. Álvarez, Carlos A. Santiviago

**Affiliations:** 1 Laboratorio de Microbiología, Departamento de Bioquímica y Biología Molecular, Facultad de Ciencias Químicas y Farmacéuticas, Universidad de Chile, 92101 Santiago, Chile; fernandez.oyarzun.paulina@gmail.com (P.A.F.); maarce.z96@gmail.com (M.Z.); j.ortb2@gmail.com (J.O.); constanza.morgado.ruiz@gmail.com (C.M.); fernando.amaya@ug.uchile.cl (F.A.); gabriel.vera@ug.uchile.cl (G.V.); carolina.rubilar@ug.uchile.cl (C.R.); beatriz.salas.v@gmail.com (B.S.); kamo.valenzuela@gmail.com (C.V.); fernando.baisonolmo@gmail.com (F.B.-O.); salvarez@uchile.cl (S.A.Á.); 2 Facultad de Medicina y Ciencia, Universidad San Sebastián, 92101 Santiago, Chile; victorcuevase@gmail.com; 3 Dynamics of Host-Pathogen Interactions Unit, Institut Pasteur, 75015 Paris, France

**Keywords:** *Salmonella*, T3SS, SPI-1, SPI-2, effector, translational fusion, *cyaA*’, adenylate cyclase, translocation, secretion

## Abstract

The type III secretion systems (T3SS) encoded in pathogenicity islands SPI-1 and SPI-2 are key virulence factors of *Salmonella*. These systems translocate proteins known as effectors into eukaryotic cells during infection. To characterize the functionality of T3SS effectors, gene fusions to the *CyaA’* reporter of *Bordetella pertussis* are often used. *CyaA’* is a calmodulin-dependent adenylate cyclase that is only active within eukaryotic cells. Thus, the translocation of an effector fused to *CyaA’* can be evaluated by measuring cAMP levels in infected cells. Here, we report the construction of plasmids pCyaA’-Kan and pCyaA’-Cam, which contain the ORF encoding *CyaA’* adjacent to a cassette that confers resistance to kanamycin or chloramphenicol, respectively, flanked by Flp recombinase target (FRT) sites. A PCR product from pCyaA’-Kan or pCyaA’-Cam containing these genetic elements can be introduced into the bacterial chromosome to generate gene fusions by homologous recombination using the Red recombination system from bacteriophage λ. Subsequently, the resistance cassette can be removed by recombination between the FRT sites using the Flp recombinase. As a proof of concept, the plasmids pCyaA’-Kan and pCyaA’-Cam were used to generate unmarked chromosomal fusions of 10 T3SS effectors to *CyaA’* in *S*. Typhimurium. Each fusion protein was detected by Western blot using an anti-CyaA’ monoclonal antibody when the corresponding mutant strain was grown under conditions that induce the expression of the native gene. In addition, T3SS-1-dependent secretion of fusion protein SipA-CyaA’ during in vitro growth was verified by Western blot analysis of culture supernatants. Finally, efficient translocation of SipA-CyaA’ into HeLa cells was evidenced by increased intracellular cAMP levels at different times of infection. Therefore, the plasmids pCyaA’-Kan and pCyaA’-Cam can be used to generate unmarked chromosomal *cyaA*’ translational fusion to study regulated expression, secretion and translocation of *Salmonella* T3SS effectors into eukaryotic cells.

## 1. Introduction

*Salmonella enterica* comprises over 2500 serotypes that are able to infect a wide range of animal hosts causing a variety of diseases ranging from gastroenteritis to systemic infections [1,2]. During the course of infection, *S. enterica* injects effector proteins into the cytoplasm of host cells using type III secretion systems (T3SS). This process is relevant for *Salmonella* virulence, as most T3SS effectors subvert host cellular functions through their enzymatic activities and physical interactions, promoting bacterial survival and colonization (reviewed in [2]). *S. enterica* harbors two independent T3SS encoded in pathogenicity islands SPI-1 and SPI-2 (T3SS-1 and T3SS-2, respectively). At least 7 effectors are known to be secreted through T3SSI-1, 22 through T3SS-2, and 9 through both systems (reviewed in [3]). Effectors include a signal sequence at the N-terminal region (first 20–30 residues) required for secretion through T3SS. These sequences lack a discernible consensus, which hinders the identification of possible effector proteins [4].

The analysis of translational fusions has proven to be very useful to evaluate effector translocation into eukaryotic host cells. Sory and coworkers described a technique using the catalytic adenylate cyclase domain of the bifunctional CyaA toxin from *Bordetella pertussis* (*CyaA’*) [5]. *CyaA’* is a calmodulin-dependent adenylate cyclase that catalyzes conversion of ATP into cyclic AMP (cAMP). Since calmodulin is ubiquitous in eukaryotic cells but absent in bacteria, the translocation of an effector fused to *CyaA’* can be evaluated by measuring the cAMP levels in infected cells.

Recently, Ramos-Morales and coworkers developed a protocol to generate site-specific *cyaA’* translational fusions in the chromosome of *S. enterica* [6] based on the Red recombination system from bacteriophage λ [7]. Although useful, this method presents an important limitation: because of the structure of the mutant allele encoding each fusion, undesirable polar effects may arise from antibiotic resistance gene expression. This is particularly relevant when genes encoding effector proteins are located in operons or nearby genes or operons encoding structural components of the associated T3SS. In this work, we describe a method that allows the generation of unmarked *cyaA’* translational fusions in the bacterial chromosome using the λ Red recombination system. To this end, we constructed template plasmids pCyaA’-Kan and pCyaA’-Cam that are used to amplify the PCR products required for recombination. As a proof of concept, we generated unmarked *cyaA’* translational fusion to genes encoding several T3SS effectors in the chromosome of *S. enterica* serovar Typhimurium (*S*. Typhimurium). The production of each fusion was evaluated by Western blot using an anti-CyaA’ monoclonal antibody, and occurred only in response to growth conditions that induce the expression of the corresponding native gene. In addition, T3SS-1-dependent secretion of a SipA-CyaA’ fusion during in vitro growth was evidenced by Western blot analysis of culture supernatants. Finally, translocation of the SipA-CyaA’ fusion into HeLa cells was confirmed by measuring cAMP levels in infected cells.

## 2. Materials and Methods

### 2.1. Bacterial Strains and Growth Conditions

The bacterial strains used in this study are listed in Table S1. All *S*. Typhimurium strains are derivatives of the wild-type, virulent strain 14028s [8,9]. Bacteria were routinely grown in Luria-Bertani (LB) medium (10 g/L tryptone, 5 g/L yeast extract, 5 g/L NaCl) at 37 °C with agitation (180 rpm). If bacteria harbored a temperature-sensitive plasmid (i.e., pKD46 or pCP20), incubations were performed at 30 °C. When required, media were supplemented with ampicillin (Amp, 100 mg/L), kanamycin (Kan, 75 mg/L), or chloramphenicol (Cam, 20 mg/L). Media were solidified by the addition of agar (15 g/L). For SPI-1-inducing conditions, bacteria were grown at 37 °C without agitation in LB medium containing 300 mM NaCl. For SPI-2-inducing conditions, bacteria were grown at 37 °C with agitation (180 rpm) in N-minimal medium (5 mM KCl, 0.5 mM (NH_4_)_2_SO_4_, 0.5 mM K_2_SO_4_, 1 mM KH_2_PO_4_, 10 μM MgCl_2_) [10] buffered in 100 mM MES (pH 5.8) and supplemented with 0.1% casamino acids and 0.4% glucose as carbon source.

### 2.2. Standard DNA Techniques

Plasmid DNA was obtained using the QIAprep Spin Miniprep kit (Qiagen, Germantown, MD, USA). PCR products were purified using the QIAquick PCR Purification kit (Qiagen, Germantown, MD, USA). DNA digestions using restriction endonucleases *Bam*HI, *Xho*I and *Spe*I (New England BioLabs, Ipswich, MA, USA) and ligations using T4 DNA ligase (New England BioLabs, Ipswich, MA, USA) were conducted as recommended by the manufacturer. When required, DNA fragments from digestions were purified from 1% agarose gels prepared in Tris-acetate-EDTA (TAE) buffer using the QIAquick Gel Extraction kit (Qiagen, Germantown, MD, USA). DNA samples were routinely analyzed by electrophoresis in 1% agarose gels prepared in TAE buffer and visualized under UV light after GelRed (Biotium Inc., Fremont, CA, USA) staining. Primers used in this study are listed in Table S2.

### 2.3. Construction of Plasmids pCyaA’-Kan and pCyaA’-Cam

To generate pCyaA’-Kan, the *cyaA’* region was amplified from pUTmini-Tn*5cyaA’* [6] using primers cyaA(F)-BamHI and cyaA(R)-XhoI (Table S2), and the PCR product was cloned into pGEM-T Easy (Promega, Madison, WI, USA) as recommended by the manufacturer to generate pGEM-T::*cyaA’*. The Kan resistance cassette flanked by Flp recombinase target (FRT) sites was amplified from pCLF4 (GenBank EU629214) [11] using primers pCLF4(F)-XhoI and pCLF4(R)-BamHI-XhoI (Table S2), and the PCR product was cloned into pGEM-T Easy as recommended by the manufacturer to generate pGEM-T::Kan. A DNA fragment containing the Kan resistance cassette flanked by FRT sites was obtained by digestion of pGEM-T::Kan with *Xho*I, and cloned into the unique *Xho*I site downstream of *cyaA’* in pGEM-T::*cyaA’* to generate pGEM-T::*cyaA’*-Kan. The orientation of the insert in the resulting plasmid was checked by PCR using primers cyaA(F)-BamHI and pCLF4(R)-BamHI-XhoI (Table S2). A DNA fragment containing *ori*R6K and *bla* gene was amplified from pKD4 (GenBank A Y048743) [7] using primers pCLF4(F)-BamHI and pCLF4(R)-BamHI (Table S2). The PCR product was purified and digested with *Bam*HI. Finally, this fragment was ligated to a fragment containing *cyaA’* and the Kan resistance cassette flanked by FRT sites obtained by digestion of pGEM-T::*cyaA’*-Kan with *Bam*HI.

To generate pCyaA’-Cam, the backbone of pCLF2 (GenBank HM047089) was amplified using primers pCLF4(F)-XhoI and pCLF4(R)-SpeI (Table S2) to incorporate unique *Xho*I and *Spe*I sites. The PCR product was purified, digested with *Xho*I and *Spe*I, and ligated to a DNA fragment containing *cyaA’* obtained by digestion of pGEM-T::*cyaA’* with *Xho*I and *Spe*I.

Plasmids pCyaA’-Kan and pCyaA’-Cam carry the R6Kγ replication origin, which requires the *trans*-acting π protein (encoded by *pir*) for replication. So, they were propagated in *Escherichia coli* DH5α λ*pir*. Derivatives of plasmid pGEM-T Easy were propagated in *E. coli* DH5α.

### 2.4. Generation of cyaA’ Translational Fusions

Derivatives of *S*. Typhimurium 14028s containing chromosomal fusions of *cyaA’* to genes encoding effectors secreted by T3SS-1 (*sipA*, *sptP,* and *sopB*), T3SS-2 (*sifA*, *sseJ*, *sopD2*, *steC*, and *sseG*), or both T3SS-1 and T3SS-2 (*spvB* and *gtgE*) were constructed by the Red-swap recombination method [7], with modifications. Briefly, a DNA fragment including the *cyaA’* ORF and a Kan resistance cassette was amplified from plasmid pCyaA’-Kan using specific primers (“xxx_H1 + C1” and “xxx_H2 + C2”) designed for each fusion (Table S2). Alternatively, the same primers were used to amplify a DNA fragment including the *cyaA’* ORF and a Cam resistance cassette from plasmid pCyaA’-Cam. *S*. Typhimurium 14028s carrying the temperature-sensitive plasmid pKD46, which expresses the λ Red recombinase system, was grown to an OD_600_ of 0.5 at 30 °C in LB medium supplemented with Amp and L-arabinose (10 mM). Bacteria were made electrocompetent by sequential washes with ice-cold sterile 15% glycerol, and transformed with ~500 ng of each PCR product. Transformants were selected on LB agar supplemented with Kan or Cam at 37 °C. The presence of each chromosomal fusion was confirmed by PCR amplification using specific “forward” primers (“xxx_Out5”) designed for each effector together with “reverse” primer CyaRev that hybridizes within *cyaA’* (Table S2).

To obtain non-polar unmarked *cyaA’* translational fusions, the corresponding antibiotic resistance cassette was removed by transforming each mutant with the temperature-sensitive plasmid pCP20, which encodes the Flp recombinase [7,12]. Transformants were selected on LB agar supplemented with Amp at 30 °C. Next, individual colonies were replica-plated on LB agar, LB agar supplemented with Amp, and LB agar supplemented with Kan or Cam, and incubated at 37 °C. Transformants that had lost pCP20 and the corresponding antibiotic resistance cassette were identified as those unable to grow in the presence of Amp and Kan or Cam. The absence of the antibiotic resistance cassette was confirmed by PCR amplification using primers cyaA(F)-BamHI and pCLF4(R)-BamHI-XhoI (Table S2). Finally, phage P22 HT105-1 *int*-201 was used to transduce mutant alleles ∆*invA*::Kan and ∆*ssaD*::Kan into a derivative of *S*. Typhimurium 14028s harboring an unmarked sipA-cyaA’ chromosomal fusion to inactive T3SS-1 or T3SS-2, respectively. The presence of each mutant allele was confirmed by PCR amplification using primers flanking the sites of substitution (Table S2).

### 2.5. Western Blot Analyses

Different bacterial strains were grown at 37 °C for 5 h under in vitro conditions that induce the expression of SPI-1 or SPI-2 genes (10 mL cultures). For preparation of whole cell lysates, bacteria recovered from 1 mL of each culture were suspended in phosphate-buffered saline (PBS) and adjusted to an OD_600_ of 2. Next, 75 μL of each bacterial suspension was mixed with 25 μL of 4 × Laemmli sample buffer (Bio-Rad, Hercules, CA, USA) and the mix was boiled for 10 min and stored at −20 °C until further use. For preparation of secreted proteins, the supernatant from each remaining culture was passed through a 0.2-µm filter to remove residual bacteria. Proteins from the supernatants were precipitated with trichloroacetic acid (10% *v*/*v*) and washed 3 times with ice-cold acetone. The pellet was air dried and subjected to metanol-chloroform precipitation [13] to remove salts, as described [14]. The final pellet was suspended in 40 μL of 4 × Laemmli sample buffer (Bio-Rad, Hercules, CA, USA) and the mix was boiled for 10 min and stored at −20 °C until further use.

Samples of each lysate (10 μL) or preparations of secreted proteins (20 μL) were resolved by SDS-PAGE in 12% polyacrylamide gels using a Mini-Protean III system (Bio-Rad, Hercules, CA, USA). The electrophoresis was conducted at 120 V (constant) using 1× running buffer (1.44% glycine, 0.3% Tris, 0.1% SDS). Transfer of proteins from polyacrylamide gels to polyvinylidene fluoride (PVDF) membranes was performed in a Mini Trans-Blot system (Bio-Rad, Hercules, CA, USA) for 90 min at 300 mA in transfer buffer (1.44% glycine, 0.3% Tris, 20% methanol). Membranes were incubated for 2 h at room temperature in a blocking solution containing 5% BSA (Sigma-Aldrich, St. Louis, MA, USA) in Tris-buffered saline supplemented with 0.1% Tween-20 (TBST). After blocking, the membranes were incubated overnight at 4 °C with the mouse anti-CyaA’ monoclonal antibody 3D1 (Santa Cruz Biotechnology, Dallas, TX, USA) diluted in blocking solution (1:10,000) or the mouse anti-DnaK monoclonal antibody [8E2/2] ab69617 (Abcam, Cambridge, MA, USA) diluted in blocking solution (1:10,000). After three washes with TBST, the membranes were incubated for 2 h at room temperature with an anti-mouse IgG antibody conjugated with horseradish peroxidase (Cell Signaling Technology, Danvers, MA, USA) diluted in blocking solution (1:10,000). Finally, the protein bands were revealed by using the SuperSignal West Femto Maximum Sensitivity Substrate (Thermo Scientific, Waltham, MA, USA) as recommended by the manufacturer. Digital images were collected using a Dyversity 4 imaging system (Syngene, Cambridge, UK) equipped with the GeneSys 1.2.5.0 software (Syngene, Cambridge, UK).

### 2.6. Mammalian Cell Culture and Infection Assays

HeLa cells were routinely grown in Dulbecco’s modified Eagle medium (DMEM) supplemented with 10% fetal bovine serum (FBS) at 37 °C in the presence of 5% CO_2_. Monolayers for infection were prepared by seeding ~2 × 10^5^ cells per well in a 24-well plate and incubating for 18 h at 37 °C in the presence of 5% CO_2_. Prior to infection, each monolayer was washed three times with sterile PBS. Bacteria were grown overnight at 37 °C under in vitro conditions that induce SPI-1 genes, washed three times with sterile PBS, suspended in 400 µL of DMEM-FBS, and added to monolayers of HeLa cells at a multiplicity of infection (MOI) of 100 bacteria/cell. The plate was centrifuged at 200 × *g* for 5 min (to facilitate the interaction of bacteria and cells) and then incubated at 37 °C in the presence of 5% CO_2_. After 1 h of incubation, the cells were washed two times with sterile PBS and incubated for 1 h in DMEM-FBS supplemented with gentamicin (200 µg/mL) to kill extracellular bacteria. Finally, the cells were washed three times with sterile PBS and further incubated for 1, 3, or 6 h post infection in DMEM-FBS supplemented with gentamicin (20 µg/mL).

### 2.7. Intracellular cAMP Measurement

Infected cell monolayers were washed three times with sterile PBS. Next, the cells were lysed using 130 µL of 1× Sample Diluent supplied with the DetectX cAMP Direct Immunoassay kit (Arbor Assays, Ann Arbor, MI, USA). Each lysate was then transferred to a microcentrifuge tube and centrifuged at 4000× *g* for 15 min at 4 °C. The supernatant was transferred to a clean microcentrifuge tube and stored at −20 °C until further use. Finally, the cAMP levels in each sample were determined by using the DetectX cAMP Direct Immunoassay kit (Arbor Assays, Ann Arbor, MI, USA) following the manufacturer instructions.

## 3. Results

### 3.1. Rationale and Design

**Figure 1:**
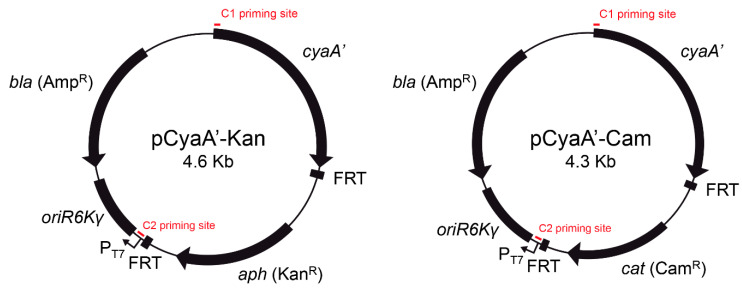
Scheme of template plasmids pCyaA’-Kan and pCyaA’-Cam for generation of unmarked chromosomal *cyaA*’ translational fusion to T3SS effectors in *Salmonella*. C1 and C2 are the priming sites for amplification of a DNA fragment including the *cyaA*’ ORF and the adjacent antibiotic resistance gene flanked by FRT sites.

Plasmids pCyaA’-Kan and pCyaA’-Cam (Figure 1) were constructed for generation of chromosomal *cyaA’* translational fusions to evaluate translocation of *S. enterica* T3SS effectors during infection of eukaryotic cells. These plasmids harbor a DNA fragment encoding the adenylate cyclase domain of the *cyaA* gene from *B. pertussis* (*cyaA’*) upstream of an antibiotic resistance cassette flanked by FRT sites (Figure 1).

**Figure 2:**
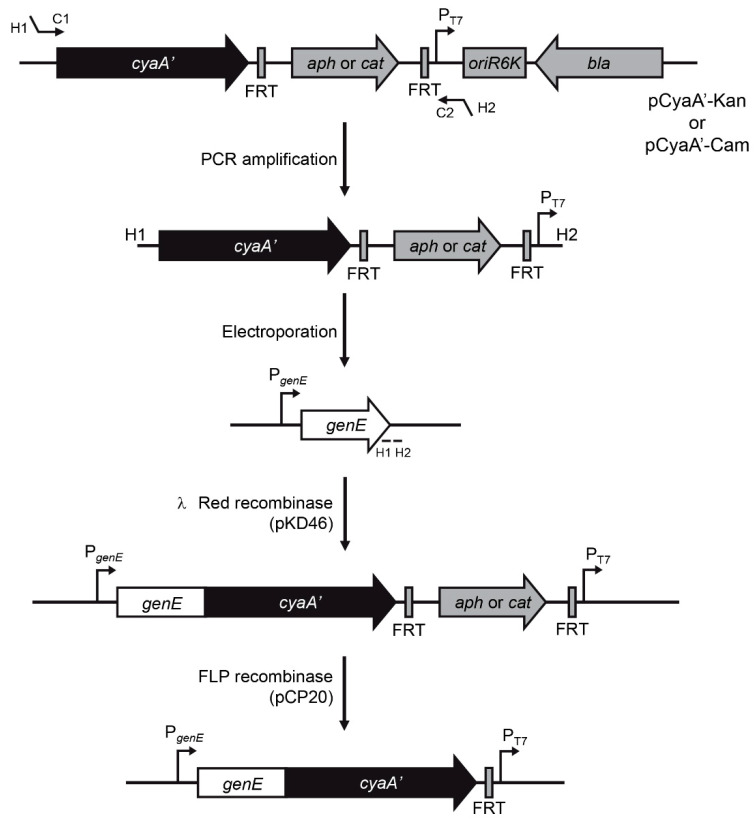
Schematic representation of the procedure to generate unmarked *cyaA’* translational fusions in the chromosome of *Salmonella*. C1 and C2 are the priming sites for amplification of a fragment of template plasmids pCyaA′-Kan and pCyaA′-Cam. H1 and H2 are specific homology regions required for insertion of the amplified fragment into a defined site in the chromosome.

To generate the translational fusions (Figure 2), a linear DNA fragment containing the *cyaA’* ORF and a Kan or a Cam resistance cassette flanked by FRT sites is obtained by PCR amplification using template plasmid pCyaA’-Kan or pCyaA’-Cam, respectively. Primers can be designed at any position in the ORF of the target chromosomal gene, although in the case of T3SS effectors it is important to maintain the signal required for secretion at the N-terminal region. We designed primers including 40-nt homology extensions (H1 and H2) and 20-nt priming sequences (C1 and C2) for pCyaA’-Kan or pCyaA’-Cam (Table S2). Extensions are homologous to regions immediately upstream (H1) and downstream (H2) of the stop codon in the target gene in order to get an in-frame translational fusion to the *cyaA’* ORF. Site-directed integration of the PCR product into the chromosome of *S*. Typhimurium is mediated by the λ Red recombination system encoded in plasmid pKD46 [7]. Finally, to eliminate possible polar effects the antibiotic resistance cassette can be removed from the chromosome by recombination between the flanking FRT sites using the Flp recombinase encoded in plasmid pCP20 [7,12].

### 3.2. Generation and Immunodetection of CyaA’ Translational Fusions to S. Typhimurium T3SS Effectors

**Figure 3:**
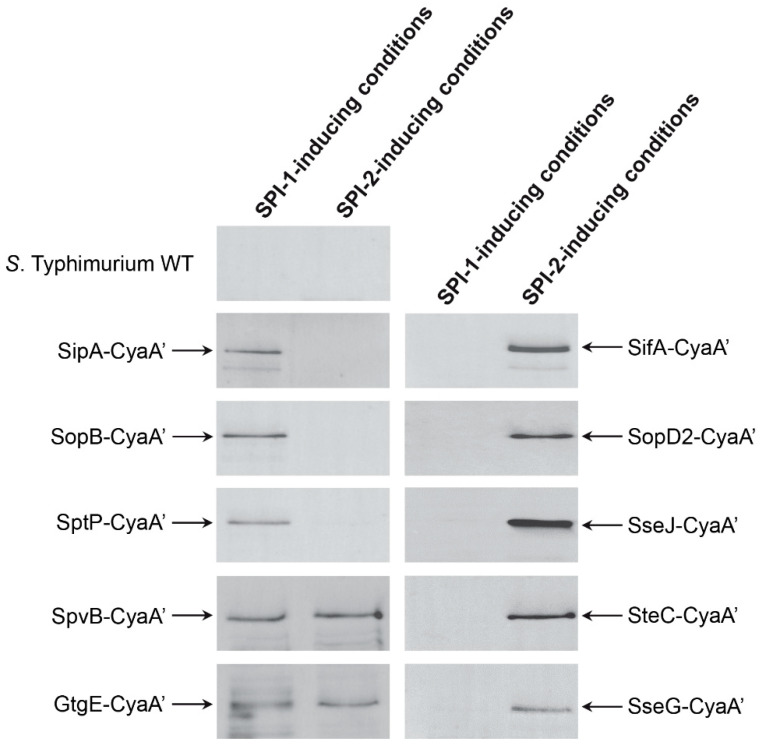
Immunodetection of *CyaA’* fusion proteins expressed by *S*. Typhimurium mutant strains grown under SPI-1- and SPI-2-inducing conditions. Bacterial strains expressing individual *CyaA’* fusion proteins were grown in vitro under conditions that induce the expression of SPI-1 genes (i.e., LB medium containing 300 mM NaCl) or SPI-2 genes (i.e., N-minimal medium adjusted to pH 5.8). Bacterial lysates prepared from each culture were subjected to SDS-PAGE in 12% polyacrylamide gels. Proteins from gels were transferred to PVDF membranes, and *CyaA’* fusion proteins were detected by Western blot using a commercial mouse anti-CyaA’ monoclonal antibody and anti-mouse IgG conjugated with horseradish peroxidase as secondary antibody.

As a proof of concept, we used pCyaA’-Kan to generate derivatives of *S*. Typhimurium 14028s harboring chromosomal *cyaA’* translational fusions to genes encoding the effector proteins SipA, SopB, SptP, SifA, SseJ, SopD2, SteC, SseG, GtgE, and SpvB. The presence of the corresponding mutant alleles was evidenced by the acquisition of the antibiotic resistance encoded in the mutagenic cassette recombined in the genome of each recipient strain, and further confirmed by PCR using primers flanking selected recombination join points (see Materials and Methods section). Next, each mutant strain generated was grown independently under culture conditions that induce the expression of genes associated with SPI-1 (i.e., LB medium containing 300 mM NaCl) or SPI-2 (i.e., N-minimal medium adjusted to pH 5.8). Lysates of bacteria recovered from each culture were subjected to SDS-PAGE and each fusion protein was detected by Western blot using a commercial anti-CyaA’ monoclonal antibody. Of note, the expression of each fusion protein was only detected when the corresponding mutant strain was grown in vitro under culture conditions that induce the expression of the native gene. Thus, fusions SipA-CyaA’, SopB-CyaA’, and SptP-CyaA’ were detected under SPI-1-inducing conditions; fusions SifA-CyaA’, SseJ-CyaA’, SopD2-CyaA’, SteC-CyaA’, and SseG-CyaA’ were detected under SPI-2-inducing conditions; and fusions GtgE-CyaA’ and SpvB-CyaA’ were detected under both SPI-1- and SPI-2-inducing conditions (Figure 3). Identical results were obtained when a mutant strain expressing SipA-CyaA’ generated using plasmid pCyaA’-Cam as template was analyzed (Figure S1). In all cases, these proteins were not detected in lysates prepared from cultures of the wild-type strain (Figure 3 and Figure S1). These observations indicate that genes encoding the fusion proteins analyzed respond to environmental cues that regulate the expression of the corresponding wild-type genes in *S*. Typhimurium.

A number of the generated fusions are encoded within SPI-1 or SPI-2; therefore, we decided to remove the antibiotic resistance cassette associated to the structure of each mutant to avoid polar effects on neighboring genes. This is particularly relevant in the case of genes located in operons, or adjacent to operons encoding regulators or structural components that are essential for T3SS-1 or T3SS-2 function. After removal of the resistance cassette, all unmarked mutant strains (including those obtained using template plasmids pCyaA’-Kan and pCyaA’-Cam) were grown under SPI-1- and SPI-2-inducing conditions and lysates of bacteria recovered from each culture were subjected to SDS-PAGE and Western blot to detect each fusion protein. As expected, all fusion proteins were detected only when the corresponding unmarked mutant strain was grown under conditions inducing the expression of the native gene. In addition, these proteins were not detected in lysates prepared from cultures of the wild-type strain (Figure S2). These results indicate that removal of the antibiotic resistance cassette does not affect the regulated expression of each fusion.

### 3.3. Secretion of a Selected CyaA’ Fusion Protein by S. Typhimurium during in Vitro Growth

**Figure 4:**
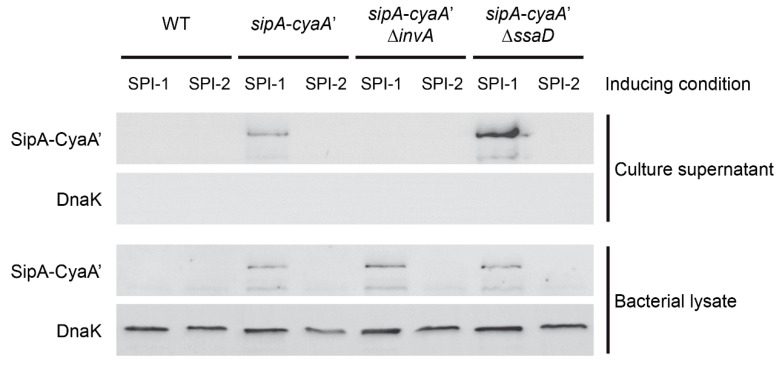
T3SS-1-dependent secretion of fusion protein SipA-CyaA’. Bacterial strains expressing SipA-CyaA’ were grown in vitro under conditions that induce the expression of SPI-1 genes (i.e., LB medium containing 300 mM NaCl) or SPI-2 genes (i.e., N-minimal medium adjusted to pH 5.8). Proteins from culture supernatants and bacterial lysates were subjected to SDS-PAGE in 12% polyacrylamide gels and transferred to PVDF membranes. SipA-CyaA’ fusion protein and DnaK were detected by Western blot using a commercial mouse anti-CyaA’ monoclonal antibody or a commercial mouse anti-DnaK monoclonal antibody. In both cases, an anti-mouse IgG conjugated with horseradish peroxidase was used as secondary antibody.

To evaluate if the fusion proteins generated by our method can be secreted to the culture medium, we analyzed culture supernatants and bacterial lysates obtained from an unmarked mutant expressing SipA-CyaA’ and derivative strains harboring mutant alleles ∆*invA*::Kan and ∆*ssaD*::Kan (to inactivate T3SS-1 and T3SS-2, respectively) grown under SPI-1- and SPI-2-inducing conditions. Samples of culture supernatants and bacterial lysates were subjected to SDS-PAGE and Western blot to detect SipA-CyaA’. As expected, the fusion protein was detected in bacterial lysates from cultures grown under SPI-1-inducing conditions, and not detected in lysates from bacteria grown under SPI-2-inducing conditions (Figure 4). On the other hand, SipA-CyaA’ was detected in culture supernatants only when bacteria harboring an active T3SS-1 were grown under SPI-1-inducing conditions (Figure 4). Of note, the cytosolic protein DnaK was not detected in culture supernatant samples, indicating that detection of SipA-CyaA’ in culture supernatants was not a consequence of bacterial lysis. Finally, SipA-CyaA’ was not detected in lysates and supernatants from cultures of the wild-type strain (Figure 4). Thus, our results indicate that *CyaA’* fusion proteins generated by our method can be secreted to the culture medium by *Salmonella* in a T3SS-dependent manner during in vitro growth.

### 3.4. Translocation of a Selected CyaA’ Fusion Protein during S. Typhimurium Infection of HeLa Cells

**Figure 5:**
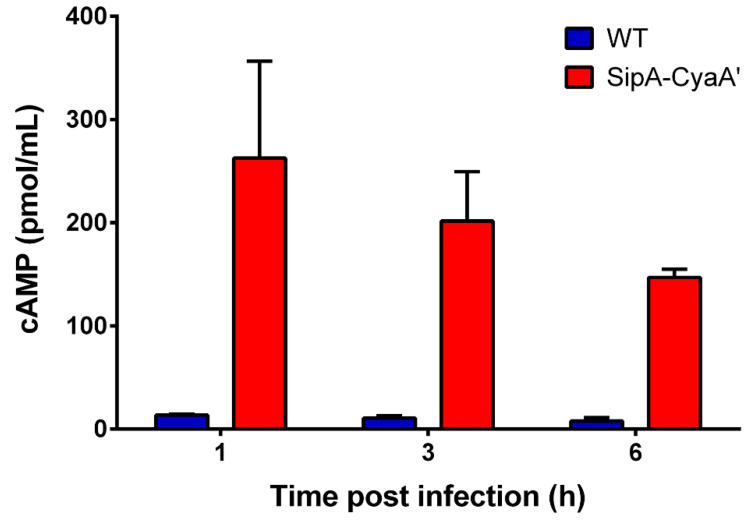
Translocation of fusion protein SipA-CyaA’ into eukaryotic cells during infection. Monolayers of HeLa cells were infected with an unmarked *S*. Typhimurium mutant strain expressing SipA-CyaA’ using a multiplicity of infection (MOI) of 100 bacteria/cell. At different times, infected cells were lysed and the level of cAMP in the lysates was determined using a commercial ELISA kit. Graph shows mean values ± SEM from an independent assay performed in duplicate.

To further evaluate if the fusion proteins generated by our method can be expressed and subsequently translocated into eukaryotic cells, we conducted in vitro infection assays using HeLa cells and our unmarked mutant expressing SipA-CyaA’. At different times of infection, the cells were lysed and the level of cAMP in the lysates was quantified using an ELISA kit (see Materials and Methods section). Efficient translocation of fusion protein SipA-CyaA’ into HeLa cells was evidenced by increased intracellular levels of cAMP at different times of infection in comparison to infections conducted with the wild-type strain (Figure 5). Thus, our results indicate that *CyaA’* fusion proteins generated by our method can be translocated by *Salmonella* into eukaryotic cells during infection.

## 4. Discussion

In the present study, we describe the construction of novel template plasmids pCyaA-Kan and pCyaA-Cam (Figure 1) for the generation of chromosomal *cyaA*’ translational fusions based on site-directed integration of a PCR product using the λ Red recombination system [7]. Unlike protocols to generate chromosomal *cyaA*’ fusions described previously [6,15], our method allows the removal of the antibiotic resistance cassette by Flp-mediated recombination [7,12], resulting in an unmarked chromosomal gene fusion (Figure 2).

Reporter gene fusions have been used as a tool to identify new effector proteins in pathogenic bacteria and for monitoring their expression, secretion, and translocation into infected cells. Several approaches involving chromosomally-encoded fusions have been developed as they are advantageous over plasmid-encoded fusions since they result in a single-copy gene fusion whose expression depends on native promoters [6,15,16,17]. In this context, *cyaA*’ has arisen as an appealing reporter gene for studying the expression, secretion, and translocation of effector proteins in the case of several pathogenic bacteria [5,15,18,19,20,21,22]. The advantage of using a *CyaA’* enzymatic tag is that the expression and secretion of the corresponding fusion protein can be detected in bacterial cultures in vitro by immunoblotting [18,21,22], and also translocation of these fusions into host cells can be monitored by its calmodulin-dependent adenylate cyclase activity, measuring the cAMP levels in infected cells [5,15,18,19,20,21,22].

Regarding *S. enterica*, a strategy based on the Red recombination system from bacteriophage λ has been successfully used for the generation of site-specific *cyaA’* translational fusions in the chromosome [6]. However, the main advantage of using our template plasmids is the possibility to generate unmarked *cyaA*’ translational fusions by removal of the antibiotic resistance cassette via Flp-mediated recombination. This novel feature is highly desirable when target genes encoding effector proteins are located in operons or nearby genes encoding structural components of the associated T3SS, where undesirable polar effects may arise from strong promoter driving the expression of the antibiotic resistance cassette.

We tested the functionality of our novel template plasmids by constructing *cyaA*’ fusions to 10 genes encoding T3SS effectors in the chromosome of *S*. Typhimurium. In all cases, the fusion protein was detected by immunoblot in bacterial lysates from cultures grown under the corresponding expression-inducing conditions (Figure 3 and Figure S1). This validates the use of *CyaA’* as an epitope tag for studying expression and regulation of genes encoding T3SS effector proteins. Of note, removal of the antibiotic resistance cassette did not affect protein detection and regulated expression of the corresponding translational fusion (Figure S2). Furthermore, using a *S*. Typhimurium strain carrying an unmarked *cyaA*’ fusion, we confirmed the T3SS-1-dependent secretion of SipA-CyaA’ during in vitro growth by Western blot analysis of culture supernatants (Figure 4). We also confirmed the translocation of SipA-CyaA’ into eukaryotic cells by measuring the levels of cAMP in HeLa cells infected with the mentioned mutant strain (Figure 5). Although not tested in this work, detection of translocated fusion proteins into eukaryotic cells could also be performed by immunoblotting after subcellular fractionation, as reported [23], which can be an attractive alternative when using host cells with elevated phosphodiesterase activity. In addition, anti-CyaA antibodies can be used to detect the intracellular localization of a given T3SS effector fused to *CyaA’* by immunofluorescence microscopy, as described [24,25].

Even though this study was focused on the characterization of *S.* Typhimurium T3SS effectors, our procedure for generation of unmarked chromosomal *cyaA*’ translational fusions can be applied to any bacterial species that uses T3SS or other secretion systems to translocate effector proteins into eukaryotic cells (e.g., T4SS and T6SS), and that allows the function of λ Red and Flp recombinases (e.g., *E. coli* and *Shigella*, among others).

## Supplementary Materials

The following are available online at https://www.mdpi.com/2076-2607/9/3/475/s1, Table S1: Bacterial strains used in this study, Table S2: Primers used in this study, Figure S1: Immunodetection of SipA-CyaA’ fusion protein expressed from a marked *S*. Typhimurium mutant strain constructed using plasmid pCyaA’-Cam as template. Figure S2: Immunodetection of *CyaA’* fusion proteins expressed from unmarked *S*. Typhimurium mutant strains grown under SPI-1- and SPI-2-inducing conditions.
