# Supplementary material for: Novel Template Plasmids pCyaA’-Kan and pCyaA’-Cam for Generation of Unmarked Chromosomal cyaA’ Translational Fusion to T3SS Effectors in Salmonella
Source: Microorganisms. 2021 Feb 25;9(3):475. doi: 10.3390/microorganisms9030475 (PMC7996335; doi:10.3390/microorganisms9030475)
Supplement: Supplementary file 1 [file microorganisms-09-00475-s001.zip › Table S1_201229.pdf]

Table S1.- Bacterial strains used in this study.

Name	Genotype or relevant characteristics	Reference or source
Salmonella enterica **serovar Typhimurium**		
WT	Wild-type, virulent strain 14028s	Laboratory collection
SipA-CyaA’ (Cam^R)	*sipA*::*cyaA*’-FRT-Cam-FRT	This study
SipA-CyaA’ (Kan^R)	*sipA*::*cyaA*’-FRT-Kan-FRT	This study
SipA-CyaA’	*sipA*::*cyaA*’-FRT	This study
SipA-CyaA’ Δ*invA*	*sipA*::*cyaA*’-FRT Δ*invA*::Kan	This study
SipA-CyaA’ Δ*ssaD*	*sipA*::*cyaA*’-FRT Δ*ssaD*::Kan	This study
SptP-CyaA’ (Kan^R)	*sptP*::*cyaA*’-FRT-Kan-FRT	This study
SptP-CyaA’	*sptP*::*cyaA*’-FRT	This study
SopB-CyaA’ (Kan^R)	*sopB*::*cyaA*’-FRT-Kan-FRT	This study
SopB-CyaA’	*sopB*::*cyaA*’-FRT	This study
SifA-CyaA’ (Kan^R)	*sifA*::*cyaA*’-FRT-Kan-FRT	This study
SifA-CyaA’	*sifA*::*cyaA*’-FRT	This study
SseJ-CyaA’ (Kan^R)	*sseJ*::*cyaA*’-FRT-Kan-FRT	This study
SseJ-CyaA’	*sseJ*::*cyaA*’-FRT	This study
SopD2-CyaA’ (Kan^R)	*sopD2*::*cyaA*’-FRT-Kan-FRT	This study
SopD2-CyaA’	*sopD2*::*cyaA*’-FRT	This study
SteC-CyaA’ (Kan^R)	*steC*::*cyaA*’-FRT-Kan-FRT	This study
SteC-CyaA’	*steC*::*cyaA*’-FRT	This study
SseG-CyaA’ (Kan^R)	*sseG*::*cyaA*’-FRT-Kan-FRT	This study
SseG-CyaA’	*sseG*::*cyaA*’-FRT	This study
SpvB-CyaA’ (Kan^R)	*spvB*::*cyaA*’-FRT-Kan-FRT	This study
SpvB-CyaA’	*spvB*::*cyaA*’-FRT	This study
GtgE-CyaA’ (Kan^R)	*gtgE*::*cyaA*’-FRT-Kan-FRT	This study
GtgE-CyaA’	*gtgE*::*cyaA*’-FRT	This study
Escherichia coli		
DH5α	Φ80*lacZΔM15* Δ(*lacZYA-argF*)*U169* *deoR phoA supE44 recA1 endA1* *hsdR17*(r_K^-, m_K^+) *thi-1 gyrA96 relA1*	Laboratory collection
DH5α λ*pir*	λ*pir* Φ80*lacZΔM15* Δ(*lacZYA-argF*)*U169* *deoR phoA supE44 recA1 endA1* *hsdR17*(r_K^-, m_K^+) *thi-1 gyrA96 relA1*	Laboratory collection
