# Supplementary material for: Novel Template Plasmids pCyaA’-Kan and pCyaA’-Cam for Generation of Unmarked Chromosomal cyaA’ Translational Fusion to T3SS Effectors in Salmonella
Source: Microorganisms. 2021 Feb 25;9(3):475. doi: 10.3390/microorganisms9030475 (PMC7996335; doi:10.3390/microorganisms9030475)
Supplement: Supplementary file 1 [file microorganisms-09-00475-s001.zip › Table S2_210201.pdf]

Table S2.- Primers used in this study.

Primer	Sequence
cyaA(F)-BamHI	CA**GGATCC**TGCAGCAATCGCATCAGGC
cyaA(R)-XhoI	TAG**CTCGAG**CTAGAACGATCGCGATCCCACC
pCLF4(F)-XhoI	ACA**CTCGAG**TGTAGGCTGGAGCTGCTTC
pCLF4(R)-BamHI-XhoI	ACA**CTCGAGGATCC**ATATGAATATCCTCCTTAG
pCLF4(F)-BamHI	TT**GGATCC**GACCATGGCTAATTCCCATGTCAGC
pCLF4(R)-BamHI	TT**GGATCC**AATCGCTCAAGACGTGTAATGCTGC
pCLF4(R)-SpeI	TT**ACTAGT**AATCGCTCAAGACGTGTAATGCTGC
sipA_(H1+C1)	TCGGGTTATTACTACCGTTGATGGCTTGCACATGCAGCGTCTGCAGCAATCGCATCAGGC
sipA_(H2+C2)	CTTCAATATCCATATTCATCGCATCTTTCCCGGTTAATTACATATGAATATCCTCCTTAG
sipA_Out5	CAGACGCTGACGCAAAAATA
sptP_(H1+C1)	AAAAGCAATGCAAGCCCAGTTGCTTATGACGACGGCAAGCCTGCAGCAATCGCATCAGGC
sptP_(H2+C2)	TACTTTCAGATAGTTCTAAAAGTAAGCTATGTTTTTATCACATATGAATATCCTCCTTAG
sptP_Out5	TGATATGTGTTCCGATGCGT
sopB_(H1+C1)	TTGGCAGTCAGTAAAAGGCATTTCTTCATTAATCACATCTCTGCAGCAATCGCATCAGGC
sopB_(H2+C2)	ACGATTTAATAGACTTTCCATATAGTTACCTCAAGACTCACATATGAATATCCTCCTTAG
sopB_Out5	AGAGACAAAAGCGGCAAAAA
sifA_(H1+C1)	TCGCTCAGAACAACAAAGCGGCTGTTTATGTTGTTTTTTACTGCAGCAATCGCATCAGGC
sifA_(H2+C2)	ACCCTGAACGTGACGTCTGAGAAAGCGTCGTCTGATTTTACATATGAATATCCTCCTTAG
sifA_Out5	ATCCGCGGTAGTCCTTCTTT
sseJ_(H1+C1)	AATGTTAGAAAGTTTTATAGCTCATCATTATTCCACTGAACTGCAGCAATCGCATCAGGC
sseJ_(H2+C2)	TGTGTTTTGCTCAAGGCGTACCGCAGCCGATGGAACTTTACATATGAATATCCTCCTTAG
sseJ_Out5	CTCACGCCAGCACACTAAAA
sopD2_(H1+C1)	ATTTATAAGTGAAAAGTCGAGTTGTCGCAATATGCTTATACTGCAGCAATCGCATCAGGC
sopD2_(H2+C2)	AAAGGCTCCATATCAGTGGGGCCTTTTTAATGACTTTTTACATATGAATATCCTCCTTAG
sopD2_Out5	GGGTTTATGGACACATTCCG
steC_(H1+C1)	GACTCTTGTGGCTAAGGTATTAAAGGATGAATTAAAAAAACTGCAGCAATCGCATCAGGC
steC_(H2+C2)	GCCCCCGGCGATTCGCAGAAAAGAACGGAACTAAATGCTACATATGAATATCCTCCTTAG
steC_Out5	CACACGGTAACGAAGTCCCT
sseG_(H1+C1)	TTTGCTGGCTCAGGTAACGCCAGAACAACGTGCGCCGGAGCTGCAGCAATCGCATCAGGC

sseG_(H2+C2)	AGAAAGCAATGAACATCCGGTATATACCTGAAAACGATTACATATGAATATCCTCCTTAG
sseG_Out5	TGATAGCGTTGCTCTTGTGG
spvB_(H1+C1)	CAGGATAAAAAGAATAATAAACATGAGGGTACTCAACTCACTGCAGCAATCGCATCAGGC
spvB_(H2+C2)	GGCCGCTCATACCACTTCTGGAATAGATTCTTAGTATCTACATATGAATATCCTCCTTAG
spvB_Out5	TCTGAGCGACGTCATTGTTC
gtgE_(H1+C1)	TTTACACTCCTCCTCCTGGAAAGACTGGTGTACCATTTTACTGCAGCAATCGCATCAGGC
gtgE_(H2+C2)	TGTTGGCGGTAGCCTGAATAATTATCTTGGTAAAGGTTAACATATGAATATCCTCCTTAG
gtgE_Out5	CCTGCAGGGAAAATCGATAA
CyaArev	CCTTGATGCCATCGAGTACG
invA_Out5	TGAGGGTTCGCTATTAACCG
invA_Out3	TGGCAATGCAAATAAATCCA
ssaD_Out5	CGGTGGTGCTAGTGGTTTTT
ssaD_Out3	GATATTGCTGCGGATCCTGT

Specific endonuclease restriction sites are indicated in bold. Underlined sequences anneal to priming site C1 or C2 in template plasmids pCyaA'-Kan and pCyaA'-Cam.
