# Supplementary material for: Novel Template Plasmids pCyaA’-Kan and pCyaA’-Cam for Generation of Unmarked Chromosomal cyaA’ Translational Fusion to T3SS Effectors in Salmonella
Source: Microorganisms. 2021 Feb 25;9(3):475. doi: 10.3390/microorganisms9030475 (PMC7996335; doi:10.3390/microorganisms9030475)
Supplement: Supplementary file 1 [file microorganisms-09-00475-s001.zip › Figure S1_210218.pdf]

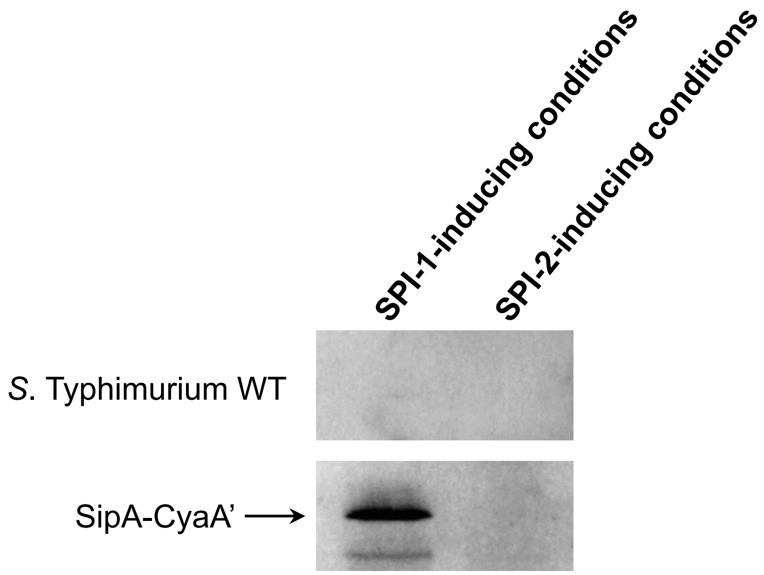

Figure S1. Immunodetection of SipA-CyaA’ fusion protein expressed from a marked *S*. Typhimurium mutant strain constructed using plasmid pCyaA’-Cam as template. A mutant strain expressing SipA-CyaA’ was grown *in vitro* under conditions that induce the expression of SPI-1 genes (i.e., LB medium containing 300 mM NaCl) or SPI-2 genes (i.e., N-minimal medium adjusted to pH 5.8). Bacterial lysates prepared from each culture were subjected to SDS-PAGE in 12% polyacrylamide gels. Proteins from polyacrylamide gels were transferred to PVDF membranes, and CyaA’ fusion proteins were detected by Western blot using a commercial mouse anti-CyaA’ monoclonal antibody and anti-mouse IgG conjugated with horseradish peroxidase as secondary antibody.
